# A prospective cohort study on the efficacy of conservative and surgical treatments for patients with ADDwoR of the temporomandibular joint

**DOI:** 10.1097/JS9.0000000000003500

**Published:** 2025-10-07

**Authors:** Pinyin Cao, Yao Liu, Jiannan Zhao, Xingyu Lyu, Xiaoqing Li, Haozhe Chen, Peng Wang, Zhan Su, Guomin Wu, Mengjie Wu, Nan Jiang, Ruiye Bi, Songsong Zhu

**Affiliations:** a State Key Laboratory of Oral Diseases and National Clinical Research Centre for Oral Diseases, Department of Orthognathic and TMJ Surgery, West China Hospital of Stomatology, Sichuan University, Chengdu, China; b State Key Laboratory of Oral Diseases, National Clinical Research Center for Oral Diseases, Department of Temporomandibular Joint, Department of Oral Anatomy and Physiology, West China Hospital of Stomatology, Sichuan University, Chengdu, China; c Department of Oral, Plastic, and Aesthetic Surgery, The Bethune Hospital of Stomatology, Jilin University, Changchun, Jilin, China; d Somatology Hospital, School of Stomatology, Zhejiang University School of Medicine, Zhejiang Provincial Clinical Research Center for Oral Diseases, Key Laboratory of Oral Biomedical Research of Zhejiang Province, Cancer Center of Zhejiang University, Hangzhou, Zhejiang, China

**Keywords:** anterior disc displacement without reduction, chewing efficiency, maximum mouth opening, pain, temporomandibular joint disorder

## Abstract

**Background:**

The treatment options for anterior disc displacement without reduction (ADDwoR) of the temporomandibular joint (TMJ) are conservative and surgical treatments; however, current therapeutic strategies for ADDwoR remain highly heterogeneous. This study aimed to evaluate the effectiveness of these two treatment options in symptom alleviation and orofacial functional restoration.

**Methods:**

The age of the patients was 18 years or older, and they underwent treatment for ADDwoR of the TMJ from December 2022 to February 2024, with patients providing 12-month follow-up data on pain, mouth-opening capacity, and chewing efficiency. The difference in the change in joint pain levels between the two methods was assessed from the baseline to the 12-month follow-up period. Mouth-opening capacity and chewing efficiency were assessed by pain-free maximum mouth opening and the TMJ chewing function self-test.

**Results:**

A total of 218 participants [mean (SD) age = 31.9 (12.9) years; 195 (89%) women] were included in this study, and the conservative treatment group and surgical treatment group included 123 individuals [mean (SD) age = 32.9 (12.7) years; 113 (92%) women] and 95 individuals [mean (SD) age = 30.6 (13.1) years; 82 (86%) women], respectively. Both the conservative treatments [mean change =− 2.45 (95% CI = −3.031 to −1.864) points] and surgical treatments [mean change =− 2.24 (95% CI −2.867 to −1.603) points] resulted in a clinically important pain reduction at 12 months. However, there was no significant difference in the effects of conservative and surgical treatments on pain reduction (*P* = 0.5737). The results of the secondary outcomes were mostly in line with those of the primary outcomes. Interestingly, among middle-aged and elderly patients with limited mouth opening, surgical treatments demonstrated a superior restoration of mouth-opening capacity compared to conservative treatments (*P* = 0.0049).

**Conclusions:**

In this cohort study of individuals with ADDwoR of the TMJ, both conservative and surgical treatments significantly improved clinically relevant pain, mouth-opening capacity and chewing efficiency in patients. And surgical intervention demonstrated superior efficacy in enhancing mouth opening among middle-aged and elderly patient.

**HIGHLIGHTS**

- This study is the first to conduct a prospective controlled study evaluating the therapeutic efficacy of conservative and surgical treatments in patients with anterior disc displacement without reduction of the temporomandibular joint.
- In this cohort study of 218 participants, both conservative treatments and surgical intervention significantly improved clinically relevant pain, mouth-opening capacity, and chewing efficiency in patients; however, no clinically relevant difference observed between the two approaches.
- In middle-aged and elderly patients with restricted mouth opening, surgical interventions showed better recovery of mouth-opening function than conservative approaches. This suggests that surgical treatment may be a more optimal choice for elderly patients.

## Introduction

Temporomandibular joint disorder (TMD) represents a substantial global health burden, with an incidence rate of approximately 31% in adults/elderly population and 11% in children/adolescents population^[1,2]^. Ranking as the second most prevalent musculoskeletal disorder following chronic low back pain, TMD leads to the joint pain, mandibular movement disorders, and discomfort in occlusion, which can really impact patients’ quality of life^[2,3]^. Anterior disc displacement (ADD) is an important manifestation of TMD, with studies showing that it occurs more than 35% in TMD patients^[1,4,5]^. Anterior disc displacement without reduction (ADDwoR) represents a severe stage in the progression of ADD, which can lead to significant facial pain, restricted mouth opening, and even facial deformity, imposing significant physiological and psychological burdens on patients[6].

Current therapeutic strategies for ADDwoR remain highly heterogeneous, with significant discrepancies in clinical practice guidelines regarding first-line intervention selection. For several decades, conservative treatments were prioritized over surgical treatments in patients with ADDwoR^[7–9]^, regardless of the long-term treatment period and the partial loss of mastication efficiency. These treatments required long-term hospital visits and adherence to a soft-food diet, significantly compromising patients’ academic performance and quality of daily life. Temporomandibular joint (TMJ) disc repositioning surgery is a surgical technique aimed at restoring the normal position of the disc through direct intervention and fixation to improve joint function and alleviate pain[10]. In adolescents of ADDwoR patients, disc repositioning surgery not only promotes condylar regeneration but also demonstrates superior regenerative condylar remodeling capacity compared to conservative modalities^[11–14]^. However, in adult patients, particularly the elderly, the condylar growth cessation and the prolonged disease history create therapeutic uncertainty, thus perpetuating clinical controversies regarding optimal treatment approaches. Traditionally, conservative treatment evaluations focused primarily on pain relief and maximum mouth opening (MMO), whereas surgical interventions emphasized radiographic changes^[9,11]^. The existence of disparate outcome assessment frameworks across therapeutic modalities significantly compromises the comparability and validity of systematic evaluations for ADDwoR treatment efficacy. We argue that symptom alleviation and functional recovery outweigh imaging outcomes, with functional recovery assessed through chewing efficiency.

To the best of our knowledge, no prospective studies have been published that directly compare the effects of conservative treatments and surgical treatments in patients with ADDwoR. Therefore, the aim of this study is to investigate the reduction in pain in patients with ADDwoR after receiving a conservative treatment versus a surgical treatment. We hope to systematically evaluate the different effects of these two treatment strategies on improving joint function, alleviating pain, and enhancing the quality of life of patients through a prospective design. We hypothesize that surgical treatments demonstrate superior therapeutic efficacy for the primary outcome (TMJ pain) in adult patients with ADDwoR compared to conservative interventions.

## Materials and methods

### Setting and design

This was a prospective single-center cohort study, which was approved by the West China Hospital of Stomatology Institutional Review Board, and all involved patients signed the informed consent form. The guidelines of the Declaration of Helsinki were followed, and all participants provided written informed consent. A total of 218 patients were examined, among whom 41 were middle-aged and elderly individuals and middle-aged and elderly individuals refers to patients aged over 45 years^[15,16]^. It was preregistered on ClinicalTrials.gov (ISRCTN99353996). Patients with ADDwoR who were diagnosed using MRI and received a conservative treatment or a surgical treatment from December 2022 to February 2024 were recruited. The inclusion criteria were as follows: age ≥18 years, ADDwoR of the TMJ, limited mouth opening, and/or pain. The exclusion criteria were as follows: a history of TMJ injury, severe systemic health conditions, extensive artefacts in MRI images that are unsuitable for measuring the disc, a history of TMJ surgery, and patients with edentulism (complete tooth loss) or functionally compromising partial edentulism (≥6 missing posterior teeth and/or ≥4 missing anterior teeth) affecting masticatory efficiency. This prospective study has been reported in line with the STROCSS guidelines[17], and it was preregistered on ClinicalTrials.gov.

### Sample calculation

To compare the treatment efficacy between the surgical and conservative treatment cohorts, the sample size was calculated by the Visual Analogue Scale (VAS) changes in the primary outcome. According to previous studies, VAS scores demonstrated a mean reduction of 3.21 ± 1.12 in the conservative treatment cohort versus 4.29 ± 1.34 in the arthrocentesis group at the 6-month follow-up[18]. Based on a two-sided *Z*-test with non-pooled variance at 90% power and α = 0.05, in additional to an estimated 20% anticipated loss rate in follow-up, the sample size was over 74 patients per group (148 participants in total). To enhance the statistical robustness of this study, the actual sample size exceeds the calculated minimum requirement.

### Outcomes

Assessments were conducted at baseline and at 12 months. The primary outcome measure was pain severity VAS, and 10 cm Visual Analogue Scale (VAS-10 cm) was used for pain evaluation. Pain levels were evaluated between 0 and 10. Secondary outcomes included pain-free MMO and masticatory ability. MMO was measured using the teeth of #11 as a reference.

Masticatory ability was being tested using Temporomandibular Joint Chewing Function Self-Test, which has 0–4 points. On a scale from 0 to 4, 0 means chewing function remains intact, and 4 means it is largely impaired. The meaning of the different points on the scale is given below:

(0) Chewing is unaffected (can eat dried beef and crack bones).

(1) Can eat normal food (peanuts, steak, etc.), with no obvious discomfort in the TMJ.

(2) Can eat typical food (steamed buns, baked cakes, stuffed pastries, etc.), with no obvious discomfort in the TMJ.

(3) Can only eat soft foods (noodles, etc.), with no obvious discomfort in the temporomandibular joint.

(4) Can only have liquid food.

### Interventions

#### Conservative treatments

For patients with ADDwoR of the TMJ with limited mouth opening and pain, injections of sodium hyaluronate are typically administered three times into the affected joint every 2 weeks. The patient sits with their head tilted towards the healthy side. The area in front of the ear is routinely disinfected, and the patient is asked to partially open their mouth, using the dip between the ear tragus and the condyle as the needle entry point. During the puncture, the needle tip is pointed forward, upward, and inward, advancing about 2–2.5 cm until it hits the bony surface of the joint, then pulling it back slightly. Once in the upper joint cavity, 2 mL of 2% lidocaine is injected to irrigate the joint. Then, all of the irrigation fluid is aspirated, and the joint cavity is irrigated 3–5 times before injecting 1 mL of sodium hyaluronate gel evenly into the upper joint cavity. The operating steps are shown in the Supplemental Digital Content Video S1, available at: http://links.lww.com/JS9/F284. For patients with ADDwoR of the TMJ that have pain, an occlusal splint should be worn for around 6 months, for about 12 hours each day. The patient is instructed to adjust the lower jaw to a comfortable position, ensuring that there is no noticeable fatigue or pain in the chewing muscles, and to note any changes in the occlusal relationship; alginate is used to make upper and lower full arch impressions and to disinfect them, and then, plaster models are cast to create an upper occlusal splint. Upon initial fitting, 20 μm occlusal paper is used to check that there is even contact between the lower full arch and the splint’s occlusal surface and to check that the condyle position in both joint discs while the splint is in place; the patient is told to wear it for about 12 hours a day, removing it during meals, and to come back for a follow-up every 4 weeks to check the occlusal contact of the splint using 20 μm occlusal paper; after 12 months, a review of clinical symptoms is conducted. All conservative treatment protocols consisted of intra-articular hyaluronic acid (HA) injections combined with 6 months of occlusal splint therapy.

#### Surgical treatments

Under general anesthesia, a modified preauricular incision was made to expose the joint capsule. The skin and subcutaneous tissue were cut while avoiding major blood vessels and branches of the facial nerve, and the upper joint cavity was accessed. The front attachment of the disc was completely released to help with the passive repositioning of the disc. We checked if the release was complete by observing the positions of the front edge of the disc, the condyle, and the joint tubercle. The TMJ disc was stitched to the back wall of the joint capsule with a commercial anchor that has two non-absorbable sutures. A week after the surgery, the patient was told to perform exercises for wide mouth opening and forward and side stretching. After 12 months, a review of clinical symptoms was conducted. Both conservative and surgical treatment groups require post-intervention rehabilitation exercises. Post-intervention rehabilitation exercises were performed by MMO training at 5 min each time, three times per day, requiring the patients to open their mouths to their maximum capacity or until achieving a width of three fingers.

#### Statistical analysis

SPSS software was used to count and analyze the survey data. The quantitative data of univariate analysis were described and counted as arithmetic mean and standard deviation, which were convenient for baseline data comparison. The distribution types of quantitative data were analyzed using the *t* test, analysis of variance, or Mann−Whitney *U* test. Qualitative data were described by the frequency/composition ratio, and the chi-square test was also performed. All tests were performed using a bilateral test, and the results were considered statistically significant when *P* < 0.05.

## Results

**Figure 1.**
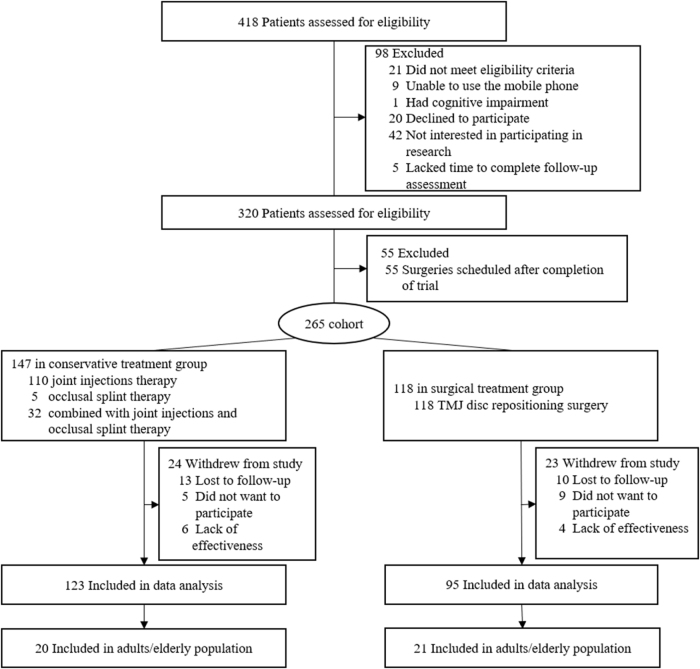
Flowchart of the inclusion process. TMJ, temporomandibular joint.

A total of 418 patients were evaluated for eligibility, and 218 patients with pain or mouth opening limitation were included in this study (Table 1). The study flow diagram is shown in Figure 1. A total of 218 participants [mean (SD) age = 31.9 (12.9) years; 195 (89%) women] were included in this study, with the conservative treatment group and surgical treatment group including 123 individuals [mean (SD) age = 32.9 (12.7) years; 113 (92%) women] and 95 individuals [mean (SD) age = 30.6 (13.1) years; 82 (86%) women], respectively. Among these 218 patients, 41 were middle-aged and elderly individuals. The mean age of the groups and the gender distribution of the patients in the groups are shown in Table 1.

**Table 1:** Baseline characteristics of the participants

	Participants, no. (%)		
Characteristic	All participants (*N* = 218)	Conservative group (*n* = 123)	Surgical group (*n* = 95)
Age, mean (SD), years	31.9 (12.9)	32.9 (12.7)	30.6 (13.1)
Sex			
Male	23 (10.6)	10 (8.1)	13 (13.7)
Female	195 (89.4)	113 (91.9)	82 (86.3)

### Effectiveness

#### Primary outcome

The mean value of VAS decreased from baseline (the beginning of the treatment) to 12 months (Table 2). Both conservative [mean change = − 2.45 (95% CI = −3.031 to −1.864; *P <* 0.0001) points] and surgical treatments [mean change = − 2.24 (95% CI = −2.867 to −1.603; *P <* 0.0001) points] resulted in a clinically important pain reduction at 12 months. However, there was no significant difference in the effects of conservative and surgical treatments on pain reduction (Table 3) (*P =* 0.5737). Furthermore, the comparison of pre- and post-treatment VAS scores between the two groups showed no statistically significant differences (Table 4).

**Table 2:** Data for clinical efficacy between baseline and follow-up (12 month) and secondary outcomes both in conservative and surgical treatments

Time/measure	Baseline mean (SE)	12 month mean (SE)	Difference between groups in change from baseline (95% CI)	*P* value^c^
VAS pain (score)				
Conservative group (*n* = 123)	3.94 (2.60)	1.50 (2.01)	−2.447 (−3.031 to −1.864)	<0.0001
Surgical group (*n* = 95)	3.40 (2.63)	1.16 (1.66)	−2.235 (−2.867 to −1.603)	<0.0001
MMO (mm)				
Conservative group (*n* = 123)	33.31 (7.19)	39.12 (5.05)	5.813 (4.732 to 6.894)	<0.0001
Surgical group (*n* = 95)	33.19 (7.83)	38.66 (5.03)	5.474 (3.753 to 7.195)	<0.0001
Masticatory ability (score)				
Conservative group (*n* = 88)	1.53 (0.93)	1.15 (0.80)	−0.4250 (−0.6718 to −0.1782)	0.0008
Surgical group (*n* = 68)	1.71 (0.99)	1.15 (0.87)	−0.5902 (−0.8923 to −0.2881)	0.0002

MMO, maximum mouth opening; VAS, Visual Analogue Scale; *, *P* value^c^ < 0.05.

**Table 3:** Data of pain reduction between baseline and follow-up (12 month) and secondary outcomes both in conservative and surgical treatments

	Mean (SE)			
Time	Conservative group (*n* = 123)	Surgical group (*n* = 95)	Difference between two groups of the variable (95% CI)	*P* value^c^
VAS pain reduction (score)	2.45 (2.61)	2.25 (2.58)	−0.1998(−0.8987 to 0.4991)	0.5737
MMO increment (mm)	5.81 (6.06)	5.47 (8.45)	−0.3393 (−2.276 to 1.598)	0.7302
Masticatory ability improvement (score)	0.37 (0.81)	0.56 (0.98)	0.1430 (−0.1335 to 0.4196)	0.3085

MMO, maximum mouth opening; VAS, Visual Analogue Scale; *, *P* value^c^ < 0.05.

**Table 4:** Data for baseline, follow-up (12 month), and change between baseline and follow-up and secondary outcomes

	Mean (SE)			
Time	Conservative group (*n* = 123)	Surgical group (*n* = 95)	Difference between groups in baseline and 12 month (95% CI)	*P* value^c^
VAS pain (score)				
Baseline	3.94 (2.60)	3.40 (2.63)	−0.5431 (−1.247 to 0.1605)	0.1296
12 month	1.50 (2.01)	1.16 (1.66)	−0.3310 (0.8349 to 0.1728)	0.1967
MMO (mm)				
Baseline	33.31 (7.19)	33.19 (7.83)	−0.1195 (−2.131 to 1.892)	0.9069
12 month	39.12 (5.05)	38.66 (5.03)	−0.4588 (−1.816 to 0.8988)	0.5060
Masticatory ability (score)				
Baseline	1.53 (0.93)	1.71 (0.99)	0.1424 (−0.1626 to 0.4473)	0.3578
12 month	1.15 (0.80)	1.15 (0.87)	0.0006684(0.2673 to 0.2660)	0.9961

MMO, maximum mouth opening; VAS, Visual Analogue Scale; *, *P* value^c^ < 0.05.

#### Secondary outcomes

**Figure 2.**
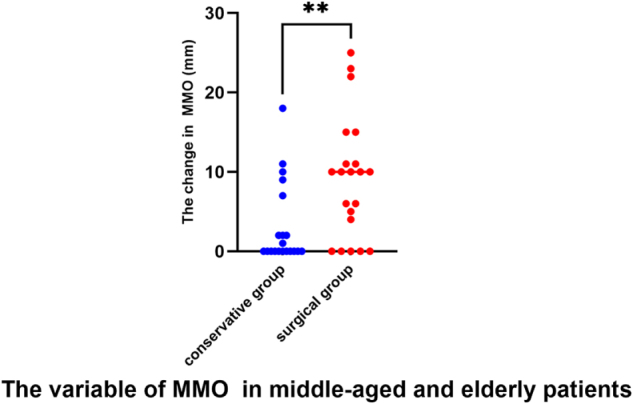
The change of MMO among middle-aged and elderly patients. Surgical treatment (21 patients) demonstrated superior restoration of mouth opening capacity compared to conservative treatment (20 patients) (*P* = 0.0049). MMO, maximum mouth opening.

A statistically significant increase was observed in MMO measurements from the baseline to the last month both in conservative [mean change = 5.81 (95% CI = 4.732 to 6.894; *P <* 0.0001) points] and surgical treatment groups [mean change = 5.47 (95% CI = 3.753 to 7.195; *P <* 0.0001) points] (Table 2). There was no statistically significant difference among the groups pertaining to the increase in assisted MMO from the baseline to the last month (Table 3). A statistically significant decrease was observed in masticatory ability from the baseline to the last month both in the conservative [mean change = −0.43 (95% CI = −0.6718 to −0.1782; *P =* 0.0008) points] and surgical treatment groups [mean change = −0.59 (95% CI = −0.8923 to −0.2881; *P =* 0.0002) points]. There was no statistically significant difference among the groups pertaining to the increase in masticatory ability from the baseline to the last month (Table 3). A comparison of pre- and post-treatment MMO and masticatory ability between the two groups revealed no statistically significant differences (Table 4). The results of the secondary outcomes were mostly in line with those of the primary outcomes. Interestingly, among middle-aged and elderly patients with limited mouth opening, surgical treatments demonstrated a superior restoration of mouth-opening capacity compared to conservative treatments (Figure. 2, *P =* 0.0049).

## Discussion

The management of ADDwoR encompasses a diverse array of therapeutic approaches, including occlusal splints[19], arthro plus intra-articular injection of HA[20], and disc repositioning with surgery[21], creating significant challenges in identifying optimal treatment strategies from the available clinical interventions. For patients with ADDwoR, researchers are endeavoring to identify the optimal therapeutic strategy by comparing the efficacy of various treatment modalities from conservative approaches to invasive approaches[22]. A randomized clinical trial study concluded that the occlusal splint treatment was superior to arthrocentesis in restoring mouth opening[18]. A retrospective study demonstrated that disc repositioning using open surgery had better short-term clinical results than stabilization splint in the treatment of ADDwoR[23]. However, no prospective published research has compared conservative and surgical treatments for patients with ADDwoR to identify the most effective method for pain reduction and mouth opening improvement. The study is the first prospective evaluation of the efficacy of conservative and surgical treatments for adult patients with ADDwoR of the TMJ. This study employed a prospective design to systematically assess patients with ADDwoR, and the results showed significant improvements in the degree to which the patients could open their mouths, and their pain levels decreased following both conservative and surgical treatments. However, there was no statistically significant difference in how well the two treatments improved mouth opening and pain. Considering the higher costs of surgical treatment and the prolonged duration of conservative therapy^[7,24]^. This finding provides an important reference for clinical practice, suggesting that treatment choices should consider the specific circumstances and treatment goals of the patients.

Our study results show that conservative treatments can effectively improve patients’ mouth opening and pain, which is consistent with the existing literature^[9,25,26]^. One of the benefits of conservative treatments is that they exhibit a low risk, making them suitable for patients with mild-to-moderate symptoms. However, for patients with more severe conditions or persistent symptoms, conservative treatments may not provide sufficient improvement^[27,28]^. Surgical treatments, particularly TMJ disc repositioning surgery, provide a good option for patients who do not respond well to conservative treatments or have more serious issues^[23,29]^. Although this study did not demonstrate a significant advantage of surgical treatments over conservative treatments in improving mouth opening and pain, surgery directly fixes the disc displacement, potentially providing more lasting benefits to patients. Moreover, we selected about 70% of the patients to study masticatory ability. A statistically significant decrease was observed in masticatory ability from the baseline to the last month, both in the conservative and surgical treatment groups. There was no statistically significant difference among the groups pertaining to the increase in masticatory ability from the baseline to the last month. This result suggests that both treatment methods exhibit positive effects on improving chewing function. Considering the mean of the points in the surgical treatment group (mean change = 0.5294) showed a higher decrease than that of the conservative group (mean change = 0.3864), individual patient differences and the complexity of conditions may account for the lack of significant difference in improving chewing function. Furthermore, we observed that patients undergoing surgery had significantly lower chewing efficiency before operation compared to those in the conservative treatment group, suggesting that surgery might be a better treatment option for patients with serious chewing problems.

Overall, the current research on TMDs predominantly focuses on adolescent and adult populations, with relatively limited investigations conducted on middle-aged and elderly patients^[6,12,30–33]^. Given that middle-aged and elderly patients with ADDwoR typically exhibit a more prolonged disease history and a greater therapeutic complexity, we conducted a focused subgroup analysis on these populations. It is particularly noteworthy that, in middle-aged and elderly patients with restricted mouth opening, surgical treatments demonstrate superior efficacy in restoring MMO. This advantage may be attributed to the fact that older patients typically present with a longer disease duration, during which intra-articular adhesions may develop[34]. Additionally, elderly patients with ADDwoR exhibit more severe morphological deformations of the articular disc (e.g., folding or thickening), which further exacerbates mechanical impingement[4]. Surgical intervention can effectively treat these adhesions within the joint cavity, and unfolding the folded and deformed articular discs restores their normal morphology, thereby facilitating the restoration of MMO. Thus, future research should further explore the long-term effects and indications of different treatment methods to offer better and more personalized treatment options for people with ADDwoR. Additionally, clinical practice should pay attention to individual patient differences and develop the most appropriate treatment plans to maximize improvements in patients’ quality of life. By comprehensively assessing patients’ conditions, expectations, and treatment responses, doctors can better formulate individualized treatment strategies, ensuring that patients achieve optimal functional recovery and enhancement in quality of life after treatment.

### Limitations

This study has several limitations when evaluating the effectiveness of conservative treatments versus surgical treatments for patients with ADDwoR of the TMJ. First, this single-center cohort study utilized patients from restricted geographic area, making it difficult to fully assess the treatment effects across different regions and populations. Moreover, the absence of randomization in this controlled clinical trial may compromise the methodological rigor and credibility of research conclusions. Therefore, the multi-center randomized trials are awaiting to be carried out to enhance sample diversity, representativeness, and the robustness of statistical results. Second, the sample size in this study is relatively small, with only 218 patients in total in both the conservative treatment group and the surgical treatment group. This sample size might not be enough to reveal subtle differences in treatment effects; thus, future research needs to expand the sample size to enhance the statistical significance and reliability of the results. Third, the study duration is not very long. TMDs are chronic conditions that are prone to recurrence. The observation period of this study was only 1 year, which does not allow for a thorough assessment of how well the treatments work over the long term and the chances of recurrence. Future studies should not only increase the sample size but also extend the observation period to obtain a clearer picture of the long-term effects of treatment and how it impacts patients’ quality of life. In summary, although this study provides some insights into the treatment of patients with ADDwoR of the TMJ, the aforementioned limitations need to be addressed to provide better and more useful insights for future research and clinical practice.

## Conclusions

In this study, treatment interventions for patients with ADDwoR of the TMJ over a 12-month period were correlated with clinically relevant improvements in pain, for both conservative treatments and surgical treatments. For middle-aged and elderly patients with limited mouth opening caused by ADDwoR in the TMJ, surgical treatments demonstrate superior effectiveness in improving the mouth-opening capacity compared to conservative treatments. Further studies should be performed to explore conservative and surgical treatments for the treatment of ADDwoR of the TMJ.

## Research registration unique identifying number (UIN)

This prospective study was reported in line with the STROCSS criteria. It was preregistered on ClinicalTrials.gov (ISRCTN99353996).

## Data statement

The data that support the finding of this study are available from the corresponding author upon reasonable request.

## The name of the trial registry and the registration number/identifier of the trial

The name: Clinical trial comparing surgical and conservative treatment in patients with anterior temporomandibular disc displacement without reduction.

Registration number: ISRCTN18007970.
